# Meta-analysis of trials comparing anastrozole and tamoxifen for adjuvant treatment of postmenopausal women with early breast cancer

**DOI:** 10.1186/1745-6215-9-47

**Published:** 2008-07-29

**Authors:** Adnan Aydiner, Faruk Tas

**Affiliations:** 1 Istanbul University, Istanbul Medical School, Department of Medical Oncology, Istanbul, Turkey

## Abstract

**Objective:**

It was aimed to review the literature and make a meta-analysis of the trials on both upfront, switching, and sequencing anastrozole in the adjuvant treatment of early breast cancer.

**Methods:**

The PubMed, ClinicalTrials.gov and Cochrane databases were systematically reviewed for randomized-controlled trials comparing anastrozole with tamoxifen in the adjuvant treatment of early breast cancer.

**Results:**

The combined hazard rate of 4 trials for event-free survival (EFS) was 0.77 (95%CI: 0.70–0.85) (*P *< 0.0001) for patients treated with anastrozole compared with tamoxifen. In the second analysis in which only ITA, ABCSG 8, and ARNO 95 trials were included and ATAC (upfront trial) was excluded, combined hazard rate for EFS was 0.64 (95%CI: 0.52–0.79) (*P *< 0.0001). In the third analysis including hazard rate for recurrence-free survival (excluding non-disease related deaths) of estrogen receptor-positive patients for ATAC trial and hazard rate for EFS of all patients for the rest of the trials, combined hazard rate was 0.73 (95%CI: 0.65–0.81) (*P *< 0.0001).

**Conclusion:**

Anastrozole appears to have superior efficacy than tamoxifen in the adjuvant hormonal treatment of early breast cancer. Until further clinical evidence comes up, aromatase inhibitors should be the initial hormonal therapy in postmenopausal early breast cancer patients and switching should only be considered for patients who are currently receiving tamoxifen.

## Introduction

The standard treatment of early breast cancer is surgery, with or without radiotherapy and chemotherapy, followed by hormonal therapy for women with hormone receptor-positive tumors. Although 5 years of tamoxifen has been the mainstay of adjuvant hormonal therapy for more than 20 years [1], tamoxifen is associated with treatment resistance [2] and some serious adverse events, like endometrial carcinoma and thromboembolic complications [3]. On the other hand, recent findings from large randomized trials have shown that anastrozole, a third generation aromatase inhibitor, has higher efficacy in terms of lower recurrence rates and better safety profile than tamoxifen as adjuvant therapy in early breast cancer [4-9]. Therefore, it is now widely accepted that anastrozole has a key role in management of postmenopausal women with early breast cancer. The American Society of Clinical Oncology Technology Assessment Panel recommends that 5 years of tamoxifen alone is no longer the best adjuvant treatment for postmenopausal patients with hormone-sensitive early breast cancer, and that optimal treatment should include an aromatase inhibitor to reduce the risk of tumor recurrence [10].

Although there are several studies on the advantages of anastrozole over tamoxifen as adjuvant hormonal therapy of hormone-sensitive early breast cancer, the timing and duration of anastrozole treatment remains in question. The Arimidex, Tamoxifen Alone or in Combination (ATAC) trial used an "upfront approach" where anastrozole was started as the first-line hormonal therapy [4,5,9], whereas other trials have used a "switching approach" where patients already receiving tamoxifen therapy for several years were randomized to continuing tamoxifen or switching to anastrozole [6-8]. A recent meta-analysis using individual data from 3 switch trials have demonstrated a statistically significant survival benefit in addition to disease- and event-free survival (DFS and EFS, respectively) rates [11]. In a third approach, anastrozole was prospectively sequenced after several years of tamoxifen treatment for newly diagnosed patients (sequencing approach) [12]. All approaches have shown a superior efficacy and safety of anastrozole over tamoxifen.

To address the overall impact of all three approaches, I sytematically reviewed the literature and did a meta-analysis of randomized trials on upfront, switching, and sequencing schedules of anastrozole adjuvant treatment in early breast cancer.

Four clinical trials were included in this meta-analysis: ATAC [4], Italian Tamoxifen Anastrozole (ITA) [8], Austrian Breast and Colorectal Cancer Study Group (ABCSG 8) [12], and Arimidex-Nolvadex (ARNO 95) [13] trials. The differences between upfront, switching, and sequencing approaches were discussed in the light of the findings of this analysis and the literature.

## Materials and methods

### Search strategy and selection criteria for trials

Systematic literature searches of PubMed, ClinicalTrials.gov and Cochrane databases were performed by using the keywords anastrozole (or Arimidex) and breast cancer. There was no date restriction, but the research was restricted to trials published in the English language. I reviewed the titles and abstracts of all the articles retrieved by this search. I considered randomized controlled trials to be eligible if they compared anastrozole with another agent in the adjuvant treatment of early breast cancer, with no restriction of study phase and regardless of doses or schedules. I didn't include dose escalation studies or single arm studies. When there were multiple records related to the same study, I extracted end point data from the report with the longest follow-up.

There were 3 recent meta-analyses, 2 on advanced disease [14,15] and 1 on early breast cancer [11]. Meta-analysis on early breast cancer was performed by combining the original individual data of 3 published studies [6-8] and did not include the results of the ATAC trial [4].

The conduct and reporting of meta-analysis was performed in accordance with the Quality of Reporting of Meta-analyses (QUOROM) statement [16].

### Data extraction and end points

I extracted the following data from each eligible trial: authors' names, journal, date of publication, number of patients randomized to each arm (ITT population) and analyzed per arm, median age of patients, menopausal and hormone receptor status, and treatment schedule and doses. I also recorded if there was a proper mode of randomization and allocation method described in the publication, median follow-up, median survival, drop-out rates and adverse events per arm, and definitions of EFS, DFS, and recurrence free survival (RFS). Hazard rates (HR) and confidence intervals (CI) for these end points were also extracted from the publications.

Quality of trials were assessed based on the reporting of method of randomization and allocation concealment, comparability of baseline treatment groups, reasons for drop-outs and if there was any evidence of differential drop-outs between treatment arms.

### Statistical analyses

HR from Cox proportional models reported in the original publications comparing anastrozole and comparator treatment was combined. The natural logarithms of the HR were combined using general variance models, and utilizing inverse variance weights[17]. Between-study heterogeneity was assessed using chi-square based Q statistic. Data were combined by using both fixed- and random-effect models and fixed-effects model results were reported when both results were nearly identical in addition to a non-significant heterogeneity test (*P *> 0.10). For our primary analysis EFS rates from ABCSG 8, ARNO 95 and ITA trials were combined but RFS rates were used from ATAC trial because EFS rates weren't reported and RFS was the most similar end point to other trials in terms of exclusion of recurrence-free deaths. A group of sensitivity analyses were made by excluding ATAC trial from our analyses and also by analysing only hormone receptor-positive patient groups.

Publication bias could not be assessed due to small number of studies included in the analysis. All analyses were performed using STATA version 9 (College Station, TX, USA).

## Results

**Figure 1:**
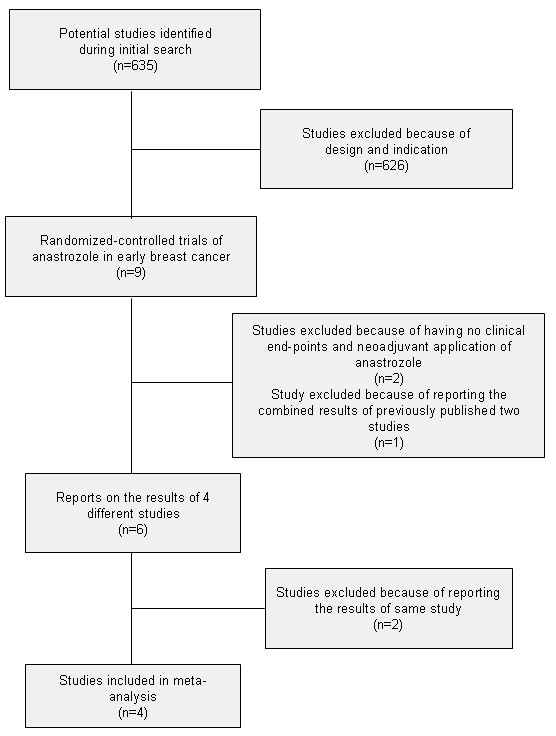
**Flow diagram for identification of trials for meta-analysis**.

I identifed 635 potential studies on anastrozole treatment in breast cancer during initial literature search (Figure. 1). Each study was evaluated for inclusion in the meta-analysis. Of these 635 studies, most were not clinical trials, but reviews and short-term laboratory studies. There were 9 randomized-controlled trials of anastrozole in patients with early breast cancer. For all these 9 trials, patients in the control group were treated with tamoxifen. Of these 9 trials, 2 were on neoadjuvant treatment and did not include clinical end points, 1 was a publication combining the results of previously published 2 studies [18]. The remaining 6 publications were the results of 4 different studies, which are ATAC [4], ITA [8], ABCSG 8 [12], and ARNO 95 [13] trials. These four trials were included in the meta-analysis.

Demographic and baseline cancer characteristics of patients and treatment regimens were similar across ITA, ABCSG 8, and ARNO 95 trials. In all these 3 trials, nearly all of the patients were estrogen receptor-positive and had received 2–3 years of tamoxifen before randomization to study groups. For both ITA and ARNO 95 trials, patients who had completed 2–3 years of adjuvant tamoxifen treatment and were relapse-free were randomized to either continue tamoxifen (20 or 30 mg/day) or switch to anastrozole (1 mg/day) for the remainder of their treatment (5 years in total). For ABCSG 8 trial, anastrozole vs. tamoxifen was prospectively sequenced after 2 years of tamoxifen treatment for newly diagnosed patients. One year after switching or sequencing, patients were clinically evaluated every 3 months in the ABCSG 8 and ITA trials; and then every 6 months or year for all 3 trials. All three studies assessed local and distant recurrence, contralateral breast cancers, and all deaths (deaths following recurrence and deaths without recurrence). The patient characteristics and treatment regimen were different for ATAC trial than the other 3 trials. ATAC trial compared 5 years of anastrozole with tamoxifen alone, or combination of the two drugs, as adjuvant therapy in postmenopausal women with localized breast cancer after surgery and chemotherapy (if applicable). Patients did not receive any hormonal treatment before randomization. Hormone receptor status for the 14% of study population was unknown at the time of randomization. It has the longest median follow-up time and highest sample size of all 4 trials (Table 1). Clinical status of patients was evaluated at 3 months, and 6 months; thereafter assessments were scheduled every 6 months up to 5 years. The summary of 4 trials is given in Table 1.

**Table 1:** Summary of trials included in meta-analysis

**Trial**	**ATAC **[4-6]	**ITA **[7,8]	**ABCSG trial** ** 8 **[9,12]	**ARNO 95** ** trial **[9,13]
**Study design**	Multicenter, randomized	Multicenter, randomized	Multicenter, randomized	Multicenter, randomized
**Patients**	Postmenopausal, receptor status +/unknown	Postmenopausal, estrogen receptor (+), received 2–3 years of tamoxifen		
**Sample size**	9366	448	2529	979
**Median follow-up (months)**	68	64	31	30
**Treatment regimen**	Upfront^a^	Switching^b^	Sequencing^c^	Switching^b^
**Estrogen receptor status**^d^				
Positive	83.7%	91.0%	99.0%	97.1%
Negative	8.3%	1.0%	1.0%	-
Unknown	8.0%	8.0%	-	2.9%
**Hazard ratios for** **event free survival ** ** (95% CI)**	All patients: 0.81 (0.73–0.91) ER positive patients: 0.76 (0.67–0.87)	0.57 (0.38–0.85)	0.68 (0.49–0.91)	0.66 (0.44–1.00)

^a^Initial treatment with anastrozole or tamoxifen alone or in combination up to 5 years

^b^Switching to anastrozole after 2–3 tamoxifen vs. continued tamoxifen for 5 years

^c^Sequencing prospectively to anastrozole vs. tamoxifen after 2 years of tamoxifen treatment

^d^For patients in anastrozole arm

**Figure 2:**
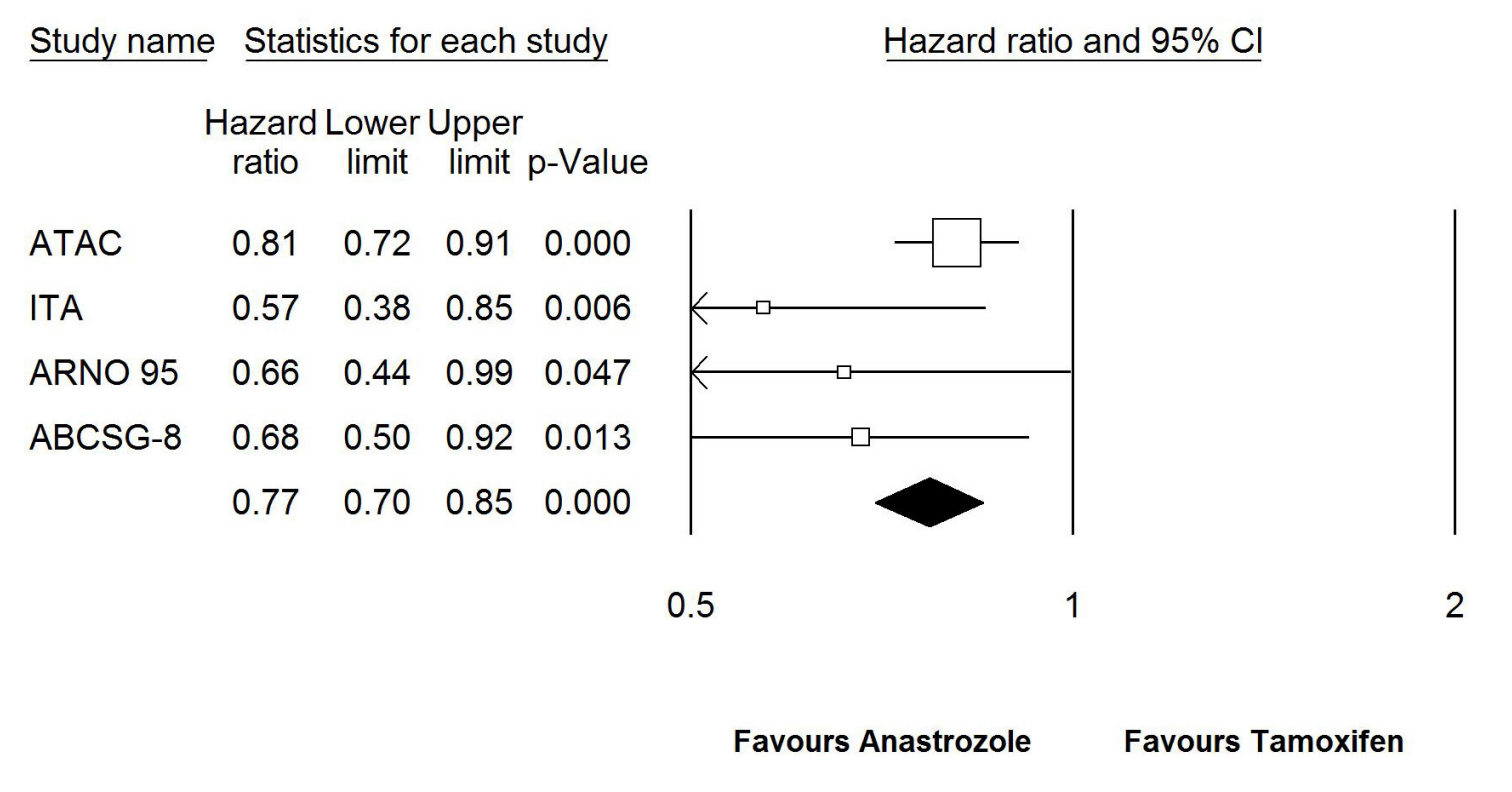
**Forest plot of event free survival for anastrozole vs. tamoxifen in fixed-effect model in which all 4 trials were included**. ^a^For ATAC trial, HR for recurrence free survival was included in the model. Test for heterogeneity (Q) = 4.128 (*P *= 0.248). HR: hazard rate, CI: confidence interval.

All studies included in this meta-analysis reported a significant improvement in EFS with anastrozole compared with tamoxifen (Table 1, Figure. 2).

**Figure 3:**
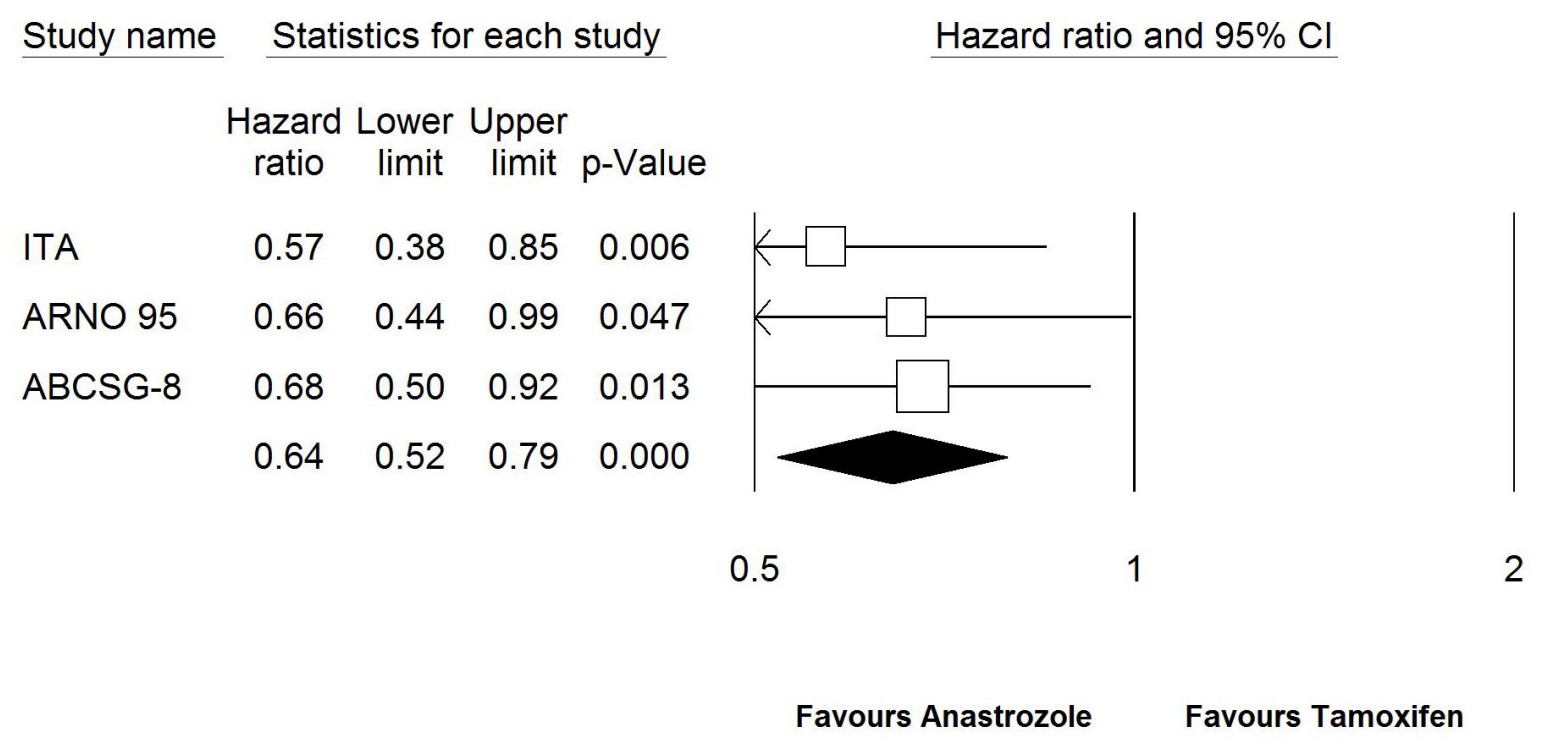
**Forest plot of event free survival for anastrozole vs. tamoxifen in fixed-effect model in which ATAC trial was excluded.** Test for heterogeneity (Q) = 0.490 (*P *= 0.783). HR: hazard rate, CI: confidence interval.

Results of our primary analysis, where HR for RFS for ATAC trial and HRs for EFS for other trials were included in the model, showed that using anastrozole as an adjuvant treatment was statistically significantly associated with increased EFS (HR: 0.77, 95%CI: 0.70–0.85, *P *< 0.0001) (Figure. 2). In a second analysis in which only switching and one sequencing trial were included and ATAC was excluded, combined HR for EFS was 0.64 (95%CI: 0.52–0.79, *P *< 0.0001) (Figure. 3). This difference between two analyses in favor of switching and sequencing trials was an expected finding due to the differences between upfront (ATAC) and other 3 trials in terms of patient population and study design as discussed in detail later.

**Figure 4:**
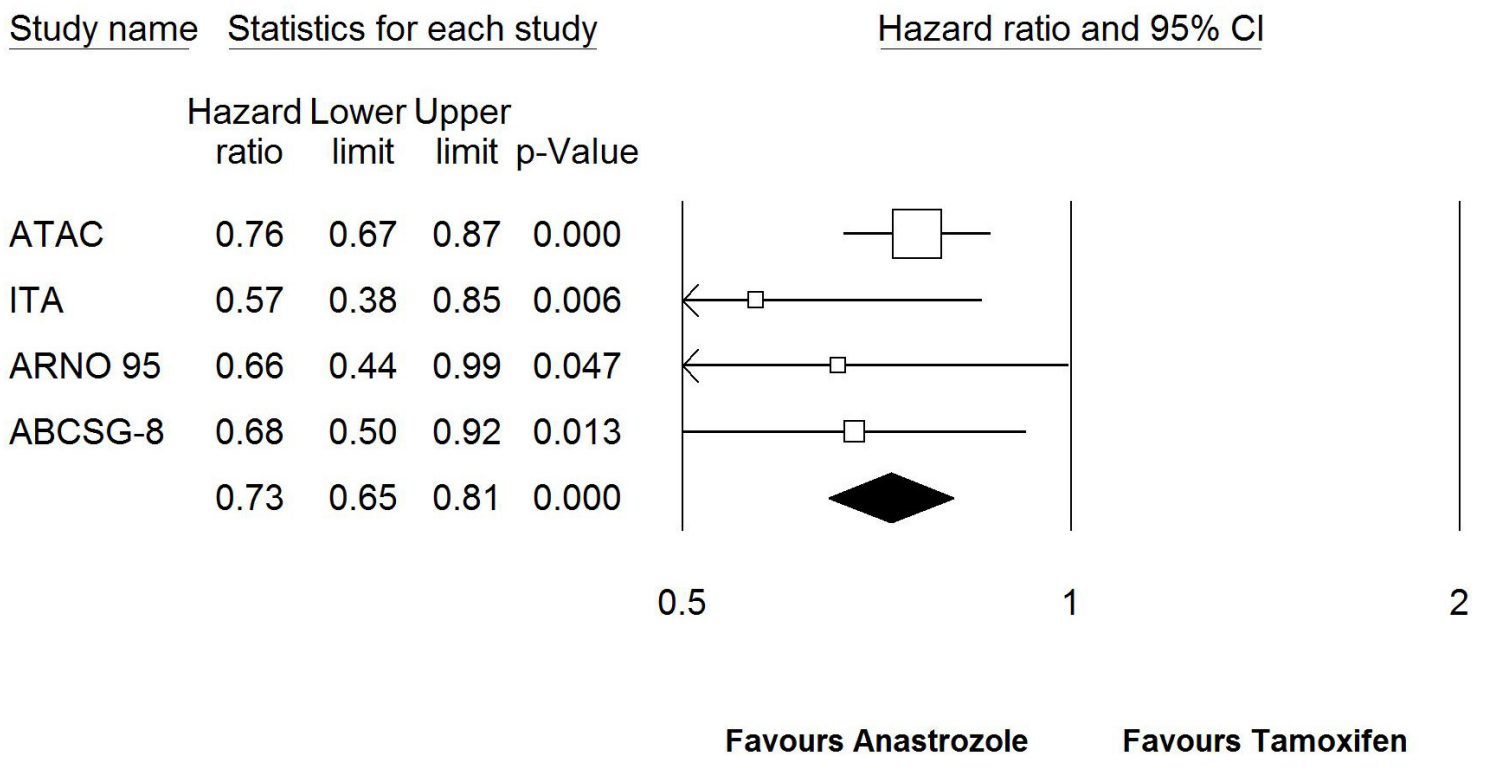
**Forest plot of event free survival for anastrozole vs. tamoxifen in fixed-effect model in which all 4 trials were included.**^a^For ATAC trial, HR for recurrence free survival of estrogen receptor positive patients was included in the model. Test for heterogeneity (Q) = 2.244 (*P *= 0.523). HR: hazard rate, CI: confidence interval.

Since in ATAC trial hormone receptor status for the 14% of study population was unknown at the time of randomization, the ratio of patients with positive estrogen receptors was lower than that of patients in switching and sequencing trials, where almost all of the patients were estrogen receptor positive (Table 1). Therefore, to maintain more similarity between trials, a third analysis was done by including HR for RFS (excluding deaths) of estrogen receptor-positive patients for ATAC trial and HRs for EFS of all patients for the other 3 the trials. The HR for RFS of estrogen receptor-positive patients for ATAC trial was 0.76 (95%CI: 0.67–0.87), *P *< 0.0001) and combined HR was found to be 0.73 (95%CI: 0.65–0.81), *P *< 0.0001) (Figure. 4).

In all 3 models, heterogeneity test revealed non-significant results.

## Discussion

The current treatment strategy for breast cancer, which is the most common cancer among women worldwide [19], includes the adjuvant use of hormonal therapy for hormone receptor-positive tumors after surgery with or without radiotherapy and chemotherapy [20]. Aromatase inhibitors have been shown to be more effective and safer than tamoxifen for adjuvant hormonal therapy of both early and advanced stage breast cancer in postmenopausal women [14,15,21-25]. Among aromatase inhibitors, anastrozole is specific to aromatase and has no significant interactions with other enzymes. Therefore, anastrozole is emerging as one of the new standards for the adjuvant treatment of hormone-sensitive early breast cancer [26]. The advantages of anastrozole over tamoxifen as an adjuvant hormonal therapy are now widely accepted, but the optimal duration of treatment and whether tamoxifen needs to be incorporated into the treatment strategy at some point remain unclear.

In this meta-analysis, the results of 4 trials were analyzed comparing anastrozole and tamoxifen in the adjuvant treatment of early breast cancer – ATAC, ITA, ABCSG 8, and ARNO 95. It was found that the combined HR for EFS was 0.77 (0.70–0.85) when all 4 trials were included in the analysis, 0.64 (0.52–0.79) when ATAC the only upfront trial was excluded, and 0.73 (0.65–0.81) when HR for RFS of estrogen receptor-positive patients in ATAC trial was included in the analysis.

In the trials comparing anastrozole and tamoxifen, anastrozole was either used as initial therapy (upfront approach) [4,5,9] or adjuvant therapy was switched to anastrozole in patients who had already received several years of tamoxifen treatment (switching approach) [6-8] or anastrozole was prospectively sequenced after several years of tamoxifen treatment for newly diagnosed patients (sequencing approach) [12] or extended adjuvant therapy with anastrozole after 5 years of tamoxifen treatment (extended treatment) [27]. Since the characteristics and disease stages of study populations were very different in these trials, it is not possible to choose the best treatment strategy just by comparing the results of these studies. The results of switching [6-8] studies appear to be better than that of the ATAC trial with the upfront approach [4,5,9]. But in the ATAC trial, hormone status for 14% of randomized patients was not known initially, patients were followed from the beginning of adjuvant treatment, and the data of all patients including those with less favorable prognoses were analyzed [4,5,9]. Another possible explanation for these findings lies in the fact that the switching trials – ITA and ARNO 95 – were performed on patients who were receiving tamoxifen for 2 or 3 years and who had no evidence of relapse. This patient population, by definition, excludes those who had relapsed during the early years of follow up, i.e., those with a less favorable prognosis were already eliminated from the cohort. Furthermore, this is the period when patients with worse prognosis (susceptible patients) are most likely to develop an event such as local or distant recurrences. This is a very well-known phenomenon in epidemiology when survival rates are not constant over time and is sometimes called "attrition of susceptibles" [6-8]. Therefore, although switching approach appears to be relatively more effective at first glance, it should not be taken as evidence for that all patients should receive several years of tamoxifen before switching to anastrozole.

It is also very important to interpret the results of switching and sequencing trials properly since the same problem of "attrition of susceptibles" appears in this comparison, too. Although study designs look similar, there is a remarkable difference between the two approaches. In the switching approach, adjuvant therapy is switched to anastrozole in patients already under tamoxifen treatment for several years [6-8]. But in the sequencing approach, anastrozole is prospectively sequenced after several years of tamoxifen treatment for newly diagnosed patients [12]. Therefore in the sequencing approach, patients are followed from the beginning of adjuvant treatment and all recurrences are taken into account. In contrast, the switching trials may comprise a patient population with a better prognosis, as explained above.

It must be noted that ABCSG 8 trial was initiated as a sequencing trial, involving upfront randomization to tamoxifen or anastrozole after 2 years of tamoxifen treatment [12]. However, in the final report, the first two years of follow-up and patients who relapsed, died or discontinued treatment were discarded and the study was transformed into a switch trial. This way, the design was similar to that of ARNO 95 and results could be combined into a single report [6]. However, this report states that out of the 3901 patients who were initially randomised, only 2262 were eligible for the combined analysis. Twenty-three (0.6%) of the ineligible patients died of causes other than breast cancer, 53 (1.4%) breast cancer-related events were encountered, 44 (1.1%) secondary carcinomas emerged and 275 (7.0%) patients had to discontinue tamoxifen for other reasons. The exclusion of these patients has, at least in part, contributed to a seemingly more favorable outcome with the switch regimen. In the combined analysis of ABCSG 8 and ARNO 95 trials in which recurrences before sequencing in ABCSG 8 trial were excluded in analysis, HR for EFS was found to be 0.60 (0.44–0.81, *P *= 0.0009), despite the higher HR observed in ARNO 95 (HR = 0.66 [0.44–0.99]) [6]. When ABCSG 8 was reported as a sequencing trial, the HR was slightly higher (HR = 0.76 [*P *= 0.068]) [12].

Since different treatment strategies of anastrozole have not been compared in a single clinical study, statistical modeling methods were used to define the best strategy [28-30]. Two main models were reported by Burstein et al. [30] and Cuzick et al. [28,29]. The results from models depend on assumptions and end points, therefore conclusions may differ. The model of Burstein et al. found that switching from tamoxifen to an aromatase inhibitor at 2.5 years yielded superior 10-year DFS than treatment with either tamoxifen or aromatase inhibitor treatment alone. In a further analysis, this model also suggested that switching from tamoxifen to an aromatase inhibitor after 2 years seems superior for estrogen and progesterone receptor-positive tumors, whereas 5 year treatment with an aromatase inhibitor may be superior for patients with estrogen positive and progesterone negative tumors [30]. Similar results were obtained in a model by Hilsenbeck and Osborne [31]. But the model by Cuzick et al. suggests that using an aromatase inhibitor as initial adjuvant therapy is a better option than switching to an aromatase inhibitor after 2 years of tamoxifen [28,29]. The recent model by Cuzick et al. has some advantages over the model by Burstein et al. which include homogeneous end points, time-dependent recurrence rates, and consideration of carry-over effect, clinical importance of early and late recurrences, and biological interactions [28,29]. Therefore Cuzick model appears more realistic than Burstein model.

In a meta-analysis combining the data of these 3 switching trials, patients who switched to anastrozole had significant improvement in EFS (HR 0.55 [0.42–0.71]; *P *< 0.0001) over those who remained on tamoxifen [11]. Due to the above-mentioned reasons, the HR for EFS in the ATAC trial was 0.79 (0.70–0.90, *P *= 0.0005), which was higher than those of each and combined data of switching trials [4]. Therefore, when ATAC data were included, the combined HR for EFS increased to 0.77 (0.70–0.85, *P *< 0.0001) when it was 0.64 (0.52–0.79, *P *< 0.0001) for the switching and sequencing trials in our analysis. Although heterogeneity test between analyzed trials revealed non-significant results for both analyses, heterogeneity was much higher in the analysis when ATAC trial was included. This may be attributed to the differences of ATAC trial from the other three trials in terms of the study design and population. To decrease the heterogeneity between the trials, a third analysis was made by including HR for RFS (excluding non-breast cancer related deaths before recurrence) of estrogen receptor-positive patients for ATAC trial and HR for EFS of all patients for the rest of the trials. In this analysis, there was remarkably less heterogeneity between trials: results of random and fixed effects models were exactly the same. The combined HR of this analysis was 0.73 (0.65–0.81, *P *< 0.0001), which was closer to the combined HR of analysis which included ITA, ARNO-95 and ABCSG-8.

This meta-analysis is important in terms of combining the results of upfront, switching, and sequencing approaches for anastrozole adjuvant treatment in early breast cancer. Our results show that anastrozole treatment improves EFS significantly, when compared to tamoxifen irrespective of the treatment approach used.

The main limitation of our meta-analysis is the low number of trials included in the analysis. To make a definitive conclusion on the optimal strategy of anastrozole adjuvant treatment in early breast cancer, trials comparing different treatment approaches – upfront, switching, sequencing, and extended treatment – would be highly desirable.

## Conclusion

As a conclusion, anastrozole appears to have superior efficacy than tamoxifen in the adjuvant hormonal treatment of early breast cancer. The question of giving anastrozole upfront or after several years of tamoxifen treatment remains open, as it is not possible to make any direct comparisons. However, it appears unlikely that giving tamoxifen, an agent that has inferior activity both in the adjuvant and metastatic setting, as first line treatment would be more effective than starting with anastrozole. This concept is also supported by several mathematical models. It is thought that, until further clinical evidence comes up, aromatase inhibitors should be the initial hormonal therapy in postmenopausal early breast cancer patients. Switching should only be considered for patients who are currently receiving tamoxifen. With the current trends, this patient population should be expected to decrease in size during the years to come, with the rare exception of premenopausal patients who become postmenopausal during tamoxifen treatment. The introduction of tamoxifen or another estrogen antagonist after the completion of aromatase inhibitor therapy may be an interesting research topic.

## List of abbreviations

ABCSG: Austrian Breast and Colorectal Cancer Study Group; ARNO: Arimidex-Nolvadex; ATAC: Arimidex, Tamoxifen Alone or in Combination; CI: Confidence interval; DFS: Disease-free survival; EFS: Event-free survival; HR: Hazard rates; ITA: Italian Tamoxifen Anastrozole; ITT: Intent-to-treat; QUOROM: Quality of Reporting of Meta-analyses ; RFS: Recurrence free survival.

## Competing interests

The authors declare that they have no competing interests.

## Authors' contributions

AA conceived of the study, and participated in its design and coordination of the statistical analysis. FT participated in the coordination and improvement of the study. All authors read and approved the final manuscript.
